# Prevalence of Testing for Diabetes Among US Adults With Overweight or Obesity, 2016–2019

**DOI:** 10.5888/pcd20.230173

**Published:** 2023-12-28

**Authors:** Yu Chen, Elizabeth A. Lundeen, Alain K. Koyama, Lyudmyla Kompaniyets, Linda J. Andes, Stephen R. Benoit, Giuseppina Imperatore, Deborah B. Rolka

**Affiliations:** 1 Division of Diabetes Translation, Centers for Disease Control and Prevention, Atlanta, Georgia; 2 Division of Nutrition, Physical Activity, and Obesity, Centers for Disease Control and Prevention, Atlanta, Georgia

## Abstract

**Introduction:**

Screening for prediabetes and type 2 diabetes may allow earlier detection, diagnosis, and treatment. The US Preventive Services Task Force recommends screening every 3 years for abnormal blood glucose among adults aged 40 to 70 years with overweight or obesity. Using IQVIA Ambulatory Electronic Medical Records, we estimated the proportion of adults aged 40 to 70 years with overweight or obesity who received blood glucose testing within 3 years from baseline in 2016.

**Methods:**

We identified 1,338,509 adults aged 40 to 70 years with overweight or obesity in 2016 and without pre-existing diabetes. We included adults whose records were present in the data set for at least 2 years before their index body mass index (BMI) in 2016 and 3 years after the index BMI (2017–2019), during which we examined the occurrence of blood glucose testing. We calculated the unadjusted and adjusted prevalence of receiving blood glucose testing.

**Results:**

The unadjusted prevalence of receiving blood glucose testing was 33.4% when it was defined as having a hemoglobin A_1c_ or fasting plasma glucose measure. The unadjusted prevalence was 74.3% when we expanded the definition of testing to include random plasma glucose and unspecified glucose measures. Adults with obesity were more likely to receive the test than those with overweight. Men (vs women) and adults aged 50 to 59 years (vs other age groups) had higher testing rates.

**Conclusion:**

Our findings could inform clinical and public health promotion efforts to improve screening for blood glucose levels among adults with overweight or obesity.

**Summary**

**What is already known on this topic?**

Screening for prediabetes and diabetes has been recommended to improve the timely provision of evidence-based diabetes prevention and treatment interventions.

**What is added by this report?**

We estimated the proportion of adults aged 40 to 70 years with overweight or obesity who received a test for blood glucose within 3 years from baseline in 2016.

**What are the implications for public health practice?**

Our findings could help inform clinical and public health efforts to improve screening for blood glucose levels among adults with overweight or obesity.

## Introduction

Screening for abnormal blood glucose levels in a clinical setting is an important strategy to detect undiagnosed diabetes and prediabetes. An estimated 37.1 million adults aged 18 years or older in the US have diabetes (14.7% of all US adults). Of those, 77.0% (28.5 million) have diagnosed diabetes and 23.0% (8.6 million) have undiagnosed diabetes (1). Moreover, approximately 96 million adults (38% of US adults) have prediabetes, but more than 80% of these adults are unaware they have the condition (1). Prediabetes is a condition in which glucose levels are higher than normal but lower than levels indicating diabetes. People with prediabetes are at increased risk of developing type 2 diabetes and tend to have high levels of health care use and expenditures (2,3). Adults who are aware of their prediabetes status are more likely than adults who are not aware of their status to engage in diabetes risk-reducing behaviors (4). Early detection of prediabetes and diabetes can improve adoption of lifestyle changes that slow the progression of diabetes and prevent diabetes complications (5).

A risk factor for abnormal blood glucose is having overweight or obesity. The 2017–2018 National Health and Nutrition Examination Survey (NHANES) found that approximately 42.5% of US adults aged 20 years or older had obesity during the 2-year survey cycle (6). Abnormal glucose metabolism is also associated with cardiovascular risk factors, such as hypertension (7). Effective blood glucose screening allows people with prediabetes and undiagnosed diabetes to be identified and referred to a type 2 diabetes primary prevention program (eg, National Diabetes Prevention Program [8]). In 2015, the US Preventive Services Task Force (USPSTF) recommended screening nonpregnant adults aged 40 to 70 years who have overweight or obesity for abnormal blood glucose every 3 years (7).

Our study aims to 1) estimate the proportion of nonpregnant adults aged 40 to 70 years with overweight or obesity who received blood glucose testing within 3 years from baseline in 2016 through 2017–2019 and 2) identify factors associated with receiving blood glucose testing.

## Methods

Data were obtained from the IQVIA (formerly known as IMS Health and Quintiles) Ambulatory Electronic Medical Records (EMR) database via the IQVIA E360 Software-as-a-Service platform (9,10). The database is one of the largest linkable, commercially available EMR databases in the industry, and it provides detailed data on clinical encounters between patients and health care providers. The database contains de-identified information recorded during outpatient encounters for a geographically diverse US patient population (9). The data are collected from more than 100,000 health care providers affiliated with more than 800 ambulatory practices and physician networks and have included more than 82 million patients since 2006 (10). We defined overweight or obesity on the basis of a body mass index (BMI) of 25.0 to 29.9 (overweight) and 30.0 or more (obesity), determined by using measured height and weight and calculated as weight in kilograms divided by height in meters squared. Receipt of a blood glucose test was defined in 2 ways: a strict USPSTF recommendation-based definition that includes having a measurement labeled as hemoglobin A_1c_ (HbA_1c_) or fasting plasma glucose (hereinafter, the “strict definition”) and a broader definition that includes measurements labeled as HbA_1c_, fasting plasma glucose, random plasma glucose, and unspecified glucose measures (hereinafter, the “broad definition”).

Our analysis identified adults aged 40 to 70 years with overweight or obesity in 2016 (the index year) and without pre-existing diabetes, defined by using a modified SUPREME-DM algorithm for EMR data (11). We included only adults whose records were present in the data set for at least 2 years before their index BMI in 2016 (to exclude those with prevalent diabetes) and 3 years after the index BMI, during which we examined the occurrence of blood glucose testing. We also excluded pregnant women from our study sample, because the USPSTF recommendation for gestational diabetes screening and treatment are different from recommendations for the general population (12). The final sample consisted of 1,338,509 adults who met the 2015 USPSTF criteria to test for abnormal blood glucose (ie, aged 40–70 years and BMI ≥25.0 kg/m^2^) (Figure).

**Figure:**
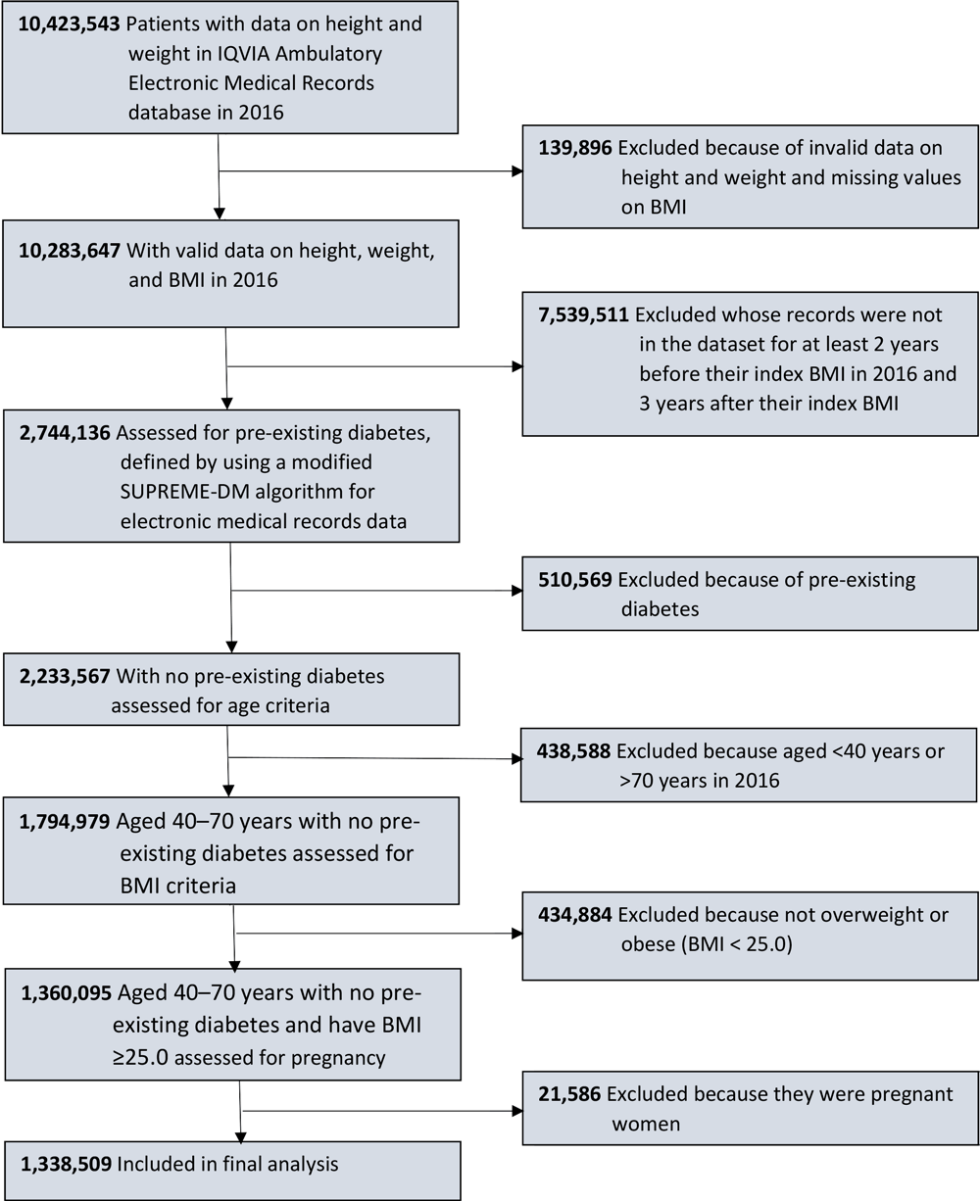
Flowchart for study sample selection. Data were obtained from the IQVIA (formerly known as IMS Health and Quintiles) Ambulatory Electronic Medical Records (EMR) database via the IQVIA E360 Software-as-a-Service platform (9,10). Abbreviation: BMI, body mass index.

### Statistical analysis

We identified factors associated with receiving blood glucose testing. These factors included sociodemographic characteristics (age, sex, race, and ethnicity), BMI, and 12 medical comorbidities in 2016 (diagnosed acute myocardial infarction, arthritis, atrial fibrillation, chronic kidney disease, chronic obstructive pulmonary disease, depression, heart failure, hyperlipidemia, hypertension, ischemic heart disease, peripheral vascular disease, and stroke). These comorbidities were chosen because of their known risk associated with type 2 diabetes (13); comorbidities were defined on the basis of the *International Classification of Diseases, 9th Revision, Clinical Modification* (ICD-9-CM) (14) and the *International Classification of Diseases, 10th Revision, Clinical Modification* (ICD-10-CM) (15) diagnosis codes in the medical record.

We calculated the unadjusted and adjusted prevalence of blood glucose testing, overall and stratified by BMI categories (overweight and obesity). The unadjusted prevalence is the crude rate of blood glucose testing. The adjusted prevalence of receiving blood glucose testing was derived from a logistic regression model (16). Specifically, in the overall sample analysis, we included BMI, age, and sex in the logistic regression model to calculate the adjusted prevalence of blood glucose testing by categories of these 3 variables. Additionally, we used BMI, age, and sex as control variables in the logistic regression models to calculate the adjusted prevalence of blood glucose testing by race and presence of each medical comorbidity. We controlled for age and sex in the logistic regressions when we calculated the adjusted prevalence of blood glucose testing stratified by BMI categories. We used SAS software version 9.4 (SAS Institute Inc) and Stata version 14 (StataCorp LLC) for all analyses.

## Results

Of the 1,338,509 adults aged 40 to 70 years who met the 2015 USPSTF criteria to test for abnormal blood glucose, nearly half had overweight (45.5%) and the other half had obesity (54.5%) (Table 1). Among the overall study sample, most patients were White (81.4%), and more than half were women (54.2%). Many patients had a diagnosis of hyperlipidemia (28.0%) or hypertension (16.7%), and a smaller percentage had a diagnosis of acute myocardial infarction (0.4%), heart failure (0.9%), or stroke (0.9%).

**Table 1:** Characteristics of the Sample of US Adults With Overweight or Obesity Who Met the 2015 USPSTF Criteria to Test for Abnormal Blood Glucose From Baseline in 2016 Through 2017–2019^a^

Characteristic	No. (%)		
	Overall	Overweight	Obesity
**Sample size**	1,338,509 (100.0)	608,681 (45.5)	729,828 (54.5)
**Age, y**			
40─49	316,535 (23.7)	133,997 (22.0)	182,538 (25.0)
50─59	470,261 (35.1)	209,416 (34.4)	260,845 (35.7)
60─70	551,713 (41.2)	265,268 (43.6)	286,445 (39.3)
**Sex**			
Female	725,977 (54.2)	313,300 (51.5)	412,677 (56.5)
Male	612,532 (45.8)	295,381 (48.5)	317,151 (43.5)
**Race and ethnicity**			
Black	101,995 (7.6)	35,732 (5.9)	66,263 (9.1)
White	1,089,989 (81.4)	499,863 (82.1)	590,126 (80.9)
Hispanic	4,197 (0.3)	1,936 (0.3)	2,261 (0.3)
Other	38,739 (2.9)	21,956 (3.6)	16,783 (2.3)
Unknown	103,589 (7.7)	49,194 (8.1)	54,395 (7.4)
**Arthritis**			
Yes	161,879 (12.1)	61,813 (10.2)	100,066 (13.7)
No	1,176,630 (87.9)	546,868 (89.8)	629,762 (86.3)
**Atrial fibrillation**			
Yes	32,586 (2.4)	12,091 (2.0)	20,495 (2.8)
No	1,305,923 (97.6)	596,590 (98.0)	709,333 (97.2)
**Acute myocardial infarction**			
Yes	5,675 (0.4)	2,477 (0.4)	3,198 (0.4)
No	1,332,834 (99.6)	606,204 (99.6)	726,630 (99.6)
**Chronic kidney disease**			
Yes	78,208 (5.8)	28,718 (4.7)	49,490 (6.8)
No	1,260,301 (94.2)	579,963 (95.3)	680,338 (93.2)
**Chronic obstructive pulmonary disease**			
Yes	66,227 (5.0)	27,086 (4.5)	39,141 (5.4)
No	1,272,282 (95.0)	581,595 (95.5)	690,687 (94.6)
**Depression**			
Yes	147,105 (11.0)	58,665 (9.6)	88,440 (12.1)
No	1,191,404 (89.0)	550,016 (90.4)	641,388 (87.9)
**Heart failure**			
Yes	12,527 (0.9)	3,871 (0.6)	8,656 (1.2)
No	1,325,982 (99.1)	604,810 (99.4)	721,172 (98.8)
**Hypertension**			
Yes	223,509 (16.7)	82,759 (13.6)	140,750 (19.3)
No	1,115,000 (83.3)	525,922 (86.4)	589,078 (80.7)
**Hyperlipidemia**			
Yes	375,323 (28.0)	166,074 (27.3)	209,249 (28.7)
No	963,186 (72.0)	442,607 (72.7)	520,579 (71.3)
**Ischemic heart disease**			
Yes	64,809 (4.8)	28,159 (4.6)	36,650 (5.0)
No	1,273,700 (95.2)	580,522 (95.4)	693,178 (95.0)
**Peripheral vascular disease**			
Yes	27,163 (2.0)	11,648 (1.9)	15,515 (2.1)
No	1,311,346 (98.0)	597,033 (98.1)	714,313 (97.9)
**Stroke**			
Yes	12,527 (0.9)	5,645 (0.9)	6,882 (0.9)
No	1,325,982 (99.1)	603,036 (99.1)	722,946 (99.1)

Abbreviation: USPSTF, US Preventive Services Task Force.

a Data were obtained from the IQVIA (formerly known as IMS Health and Quintiles) Ambulatory Electronic Medical Records (EMR) database via the IQVIA E360 Software-as-a-Service platform (9,10).

In the 3-year follow-up period, we found that 33.4% (unadjusted prevalence) of adults eligible for screening received a test for blood glucose when we used the strict definition of a blood glucose test (Table 2). Among adults with overweight, the unadjusted prevalence of blood glucose testing was 28.7%. Among adults with obesity, the unadjusted prevalence of blood glucose testing was 37.3%. When we used the broad definition of a blood glucose test, the unadjusted prevalence of receiving a blood glucose test was 74.3% among all adults eligible for screening, and among persons with overweight and obesity, the prevalence was 73.9% and 74.7%, respectively. In addition, we found a higher probability of receiving glucose testing among adults aged 50 to 59 years compared with other age groups. Men had a slightly higher testing rate than women (eg, unadjusted rate using the strict definition: 33.9% vs 32.9%). We found a higher testing rate among Black adults than among White adults. For example, the unadjusted testing rate using the strict definition was 37.0% among Black adults and 32.4% among White adults. Among the medical comorbidities, the unadjusted prevalence of receiving blood glucose testing was higher among adults who had (vs did not have) chronic kidney disease, chronic obstructive pulmonary disease, depression, hyperlipidemia, hypertension, ischemic heart disease, peripheral vascular disease, or stroke. The patterns for the adjusted prevalence of receiving blood glucose testing were similar to patterns for the unadjusted prevalence.

**Table 2:** Unadjusted and Adjusted Prevalence of Receiving Blood Glucose Testing for the Overall Study Sample (N = 1,338,509), by Definition of Blood Glucose Testing^a^

Characteristic	Strict definition of blood glucose testing^b^		Broad definition of blood glucose testing^c^	
	Unadjusted, %	Adjusted, % (95% CI)^d^	Unadjusted, %	Adjusted, % (95% CI)^d^
**Overall**	33.4	—	74.3	—
**BMI^e^**				
Overweight	28.7	28.6 (28.5–28.7)	73.9	73.9 (73.8–74.0)
Obesity	37.3	37.3 (37.2–37.4)	74.7	74.7 (74.6–74.8)
**Age, y**				
40–49	32.5	32.2 (32.1–32.4)	74.3	74.2 (74.1–74.4)
50–59	34.4	34.3 (34.2–34.4)	75.1	75.1 (75.0–75.2)
60–70	33.0	33.2 (33.1–33.4)	73.7	73.7 (73.6–73.8)
**Sex**				
Female	32.9	32.7 (32.6–32.8)	74.1	74.1 (74.0–74.2)
Male	33.9	34.2 (34.0–34.3)	74.6	74.6 (74.5–74.7)
**Race and ethnicity** f				
Black	37.0	36.2 (35.9–36.5)	71.4	71.3 (71.0–71.6)
Hispanic	—	—	—	—
White	32.4	32.4 (32.3–32.5)	74.8	74.8 (74.7–74.9)
Other	47.4	48.8 (48.3–49.3)	79.0	79.0 (78.6–79.4)
Unknown	34.9	35.1 (34.8–35.4)	70.7	70.7 (70.4–70.9)
**Arthritis**				
Yes	33.9	33.2 (33.0–33.5)	74.1	74.2 (74.0–74.4)
No	33.3	33.4 (33.3–33.5)	74.4	74.4 (74.2–74.4)
**Atrial fibrillation**				
Yes	31.4	30.4 (29.9–30.9)	75.3	75.4 (74.9–75.8)
No	33.4	33.5 (33.4–33.5)	74.3	74.3 (74.2–74.4)
**Acute myocardial infarction**				
Yes	26.6	26.0 (24.9–27.2)	64.9	64.8 (63.6–66.1)
No	33.4	33.4 (33.3–33.5)	74.4	74.4 (74.3–74.5)
**Chronic kidney disease**				
Yes	56.8	56.0 (55.7–56.4)	88.2	88.3 (88.0–88.5)
No	31.9	32.0 (31.9–32.0)	73.5	73.5 (73.4–73.6)
**Chronic obstructive pulmonary disease**				
Yes	37.1	36.7 (36.3–37.1)	78.6	78.7 (78.4–79.0)
No	33.2	33.2 (33.1–33.3)	74.1	74.1 (74.0–74.2)
**Depression**				
Yes	39.2	39.0 (38.8–39.3)	82.5	82.6 (82.4–82.8)
No	32.7	32.7 (32.6–32.8)	73.3	73.3 (73.2–73.4)
**Heart failure**				
Yes	34.1	32.7 (31.9–33.5)	78.7	78.8 (78.0–79.5)
No	33.4	33.4 (33.3–33.5)	74.3	74.3 (74.2–74.4)
**Hypertension**				
Yes	44.3	43.6 (43.4–43.8)	87.5	87.6 (87.5–87.8)
No	31.2	31.3 (31.2–31.4)	71.7	71.6 (71.5–71.7)
**Hyperlipidemia**				
Yes	44.8	44.8 (44.7–45.0)	89.7	89.9 (89.8–90.0)
No	28.9	28.9 (28.8–29.0)	68.4	68.2 (68.1–68.3)
**Ischemic heart disease**				
Yes	34.2	33.5 (33.2–34.0)	75.2	75.3 (75.0–75.7)
No	33.3	33.4 (33.3–33.5)	74.3	74.3 (74.2–74.4)
**Peripheral vascular disease**				
Yes	35.6	35.3 (34.8–35.9)	75.0	75.1 (74.6–75.6)
No	33.3	33.3 (33.2–33.4)	74.3	74.3 (74.2–74.4)
**Stroke**				
Yes	34.9	34.8 (34.0–35.6)	75.8	76.0 (75.3–76.7)
No	33.4	33.4 (33.3–33.5)	74.3	74.3 (74.2–74.4)

Abbreviations: BMI, body mass index; EMR, electronic medical record; USPSTF, US Preventive Services Task Force.

a Data were obtained from the IQVIA (formerly known as IMS Health and Quintiles) Ambulatory EMR database via the IQVIA E360 Software-as-a-Service platform (9,10).

b Based on a strict USPSTF recommendation-based definition: tests that were clearly labeled in the EMR as hemoglobin A_1c_, fasting plasma glucose, or oral glucose tolerance test. No oral glucose tolerance tests were found in the IQVIA database used for this analysis.

c Includes measurements labeled as hemoglobin A_1c_, fasting plasma glucose, random plasma glucose, and unspecified measures.

d We used logistic regression to calculate the margins which gives the adjusted prevalence of receiving blood glucose testing to control for potential confounding effects. We included BMI, age, and sex in the logistic regression model to calculate the adjusted prevalence of blood glucose testing by categories of these 3 variables. We used BMI, age, and sex as control variables in the logistic regression models to calculate the adjusted prevalence of blood glucose testing by race and presence of each medical comorbidity. We also calculated the odds ratio of receiving blood glucose testing among adults with obesity vs overweight; these data are available upon request from the corresponding author.

e Calculated as weight in kilograms divided by height in meters squared. Overweight defined as BMI from 25.0 to 29.9, obesity as BMI ≥30.0.

f Data on ethnicity (Hispanic origin) are not captured accurately in IQVIA electronic health records, and thus, these values have been suppressed.

The prevalence of receiving blood glucose testing differed by BMI category, and the overall prevalence of receiving a blood glucose test was higher among adults with obesity than among adults with overweight (Table 3). For example, using the strict definition of blood glucose testing, the unadjusted rate of having glucose testing was 36.7% among women with obesity and 27.9% among women with overweight. We found a similar pattern among other subgroups of demographic characteristics and medical comorbidities. Additionally, we found that in both groups (adults with obesity or overweight), the prevalence of receiving blood glucose testing was higher among adults aged 50 to 59 years than among other age groups and higher among men than women. Also, similar to the overall study sample, when we stratified by overweight or obesity, we found that among both groups the prevalence of receiving blood glucose testing was higher among adults who had (vs did not have) chronic kidney disease, chronic obstructive pulmonary disease, depression, hyperlipidemia, hypertension, or peripheral vascular disease.

**Table 3:** Unadjusted and Adjusted Prevalence of Receiving Blood Glucose Testing Among Study Sample (N = 1,338,509), Stratified by Body Mass Index Categories and Definition of Blood Glucose Testing^a^

Characteristic	Overweight^b^ (n = 608,681)				Obesity^c^ (n = 729,828)			
	Strict definition of blood glucose testing^d^		Broad definition of blood glucose testing^e^		Strict definition of blood glucose testing^d^		Broad definition of blood glucose testing^e^	
	Unadjusted, %	Adjusted, % (95% CI)^f^	Unadjusted, %	Adjusted, % (95% CI)^f^	Unadjusted, %	Adjusted, % (95% CI)^f^	Unadjusted, %	Adjusted, % (95% CI)^f^
**Age, y**								
40–49	26.8	26.8 (26.6–27.0)	73.2	73.3 (73.0–73.5)	36.7	36.7 (36.4–36.9)	75.1	75.2 (75.0–75.4)
50–59	29.5	29.5 (29.3–29.7)	74.7	74.7 (74.5–74.9)	38.4	38.4 (38.2–38.5)	75.5	75.5 (75.3–75.6)
60‒70	29.1	29.0 (28.9–29.2)	73.6	73.6 (73.5–73.8)	36.7	36.7 (36.5–36.9)	73.8	73.8 (73.6–74.0)
**Sex**								
Female	27.9	27.9 (27.8–28.1)	73.8	73.8 (73.6–73.9)	36.7	36.7 (36.6–36.9)	74.4	74.4 (74.2–74.5)
Male	29.5	29.5 (29.3–29.7)	74.0	73.9 (73.8–74.1)	38.0	38.0 (37.9–38.2)	75.2	75.2 (75.0–75.3)
**Race and ethnicity** g								
Black	33.4	33.6 (33.1–34.1)	70.9	71.0 (70.5–71.4)	39.0	39.1 (38.7–39.5)	71.7	71.7 (71.3–72.0)
Hispanic	—	—	—	—	—	—	—	—
White	27.3	27.3 (27.1–27.4)	74.1	74.1 (74.0–74.2)	36.7	36.6 (36.5–36.8)	75.4	75.4 (75.3–75.5)
Other	46.3	46.7 (46.1–47.4)	80.6	80.7 (80.1–81.2)	49.0	49.0 (48.3–49.8)	76.9	76.7 (76.1–77.4)
Unknown	31.2	31.3 (30.9–31.7)	70.8	70.8 (70.4–71.2)	38.2	38.2 (37.8–38.6)	70.6	70.5 (70.1–70.9)
**Arthritis**								
Yes	29.1	28.9 (28.6–29.3)	74.7	74.7 (74.4–75.0)	36.8	36.9 (36.6–37.2)	73.8	74.0 (73.8–74.3)
No	28.7	28.7 (28.5–28.8)	73.8	73.8 (73.7–73.9)	37.4	37.3 (37.2–37.5)	74.9	74.8 (74.7–74.9)
**Atrial fibrillation**								
Yes	26.5	25.8 (25.1–26.6)	75.4	75.5 (74.7–76.3)	34.4	34.3 (33.6–34.9)	75.2	75.4 (74.9–76.0)
No	28.7	28.8 (28.6–28.9)	73.8	73.8 (73.7–73.9)	37.4	37.4 (37.3–37.5)	74.7	74.7 (74.6–74.8)
**Acute myocardial infarction**								
Yes	22.9	22.3 (20.7–23.9)	63.9	63.8 (61.9–65.7)	29.5	29.2 (27.6–30.7)	65.6	65.6 (64.0–67.3)
No	28.7	28.7 (28.6–28.8)	73.9	73.9 (73.8–74.0)	37.3	37.3 (37.2–37.4)	74.8	74.8 (74.7–74.9)
**Chronic kidney disease**								
Yes	49.4	49.2 (48.6–49.8)	86.8	86.8 (86.4–87.2)	61.1	61.2 (60.8–61.7)	89.1	89.2 (88.9–89.4)
No	27.7	27.7 (27.6–27.8)	73.2	73.2 (73.1–73.3)	35.6	35.5 (35.4–35.7)	73.7	73.7 (73.6–73.8)
**Chronic obstructive pulmonary disease**								
Yes	32.1	31.9 (31.3–32.4)	78.7	78.8 (78.3–79.3)	40.7	40.7 (40.2–41.2)	78.5	78.7 (78.3–79.1)
No	28.5	28.5 (28.4–28.7)	73.6	73.6 (73.5–73.8)	37.1	37.1 (37.0–37.2)	74.5	74.5 (74.4–74.6)
**Depression**								
Yes	33.2	33.5 (33.1–33.9)	82.3	82.4 (82.1–82.7)	43.3	43.5 (43.2–43.9)	82.6	82.7 (82.5–83.0)
No	28.2	28.2 (28.1–28.3)	73.0	73.0 (72.8–73.1)	36.5	36.4 (36.3–36.5)	73.7	73.6 (73.5–73.7)
**Heart failure**								
Yes	27.2	26.6 (25.3–28.0)	78.3	78.3 (77.0–79.6)	37.3	37.2 (36.2–38.2)	78.9	79.2 (78.4–80.1)
No	28.7	28.7 (28.6–28.8)	73.8	73.8 (73.7–74.0)	37.3	37.3 (37.2–37.4)	74.7	74.7 (74.6–74.8)
**Hypertension**								
Yes	38.3	38.1 (37.8–38.4)	87.4	87.5 (87.3–87.7)	47.9	47.9 (47.7–48.2)	87.6	87.7 (87.5–87.9)
No	27.2	27.2 (27.1–27.3)	71.7	71.7 (71.6–71.8)	34.8	34.7 (34.6–34.9)	71.7	71.6 (71.5–71.7)
**Hyperlipidemia**								
Yes	39.5	39.6 (39.3–39.8)	89.9	90.1 (90.0–90.2)	49.0	49.2 (49.0–49.4)	89.5	89.7 (89.6–89.9)
No	24.6	24.6 (24.5–24.7)	67.9	67.7 (67.5–67.8)	32.6	32.5 (32.4–32.6)	68.8	68.6 (68.4–68.7)
**Ischemic heart disease**								
Yes	30.6	29.8 (29.3–30.4)	75.2	75.2 (74.7–75.7)	37.0	36.7 (36.2–37.2)	75.3	75.4 (75.0–75.9)
No	28.6	28.6 (28.5–28.8)	73.8	73.8 (73.7–73.9)	37.3	37.3 (37.2–37.4)	74.7	74.7 (74.6–74.8)
**Peripheral vascular disease**								
Yes	31.6	31.3 (30.4–32.1)	74.6	74.6 (73.8–75.4)	38.7	38.8 (38.0–39.5)	75.3	75.6 (74.9–76.3)
No	28.6	28.6 (28.5–28.8)	73.9	73.9 (73.7–74.0)	37.3	37.3 (37.1–37.4)	74.7	74.7 (74.6–74.8)
**Stroke**								
Yes	29.3	28.8 (27.7–30.0)	73.9	73.9 (72.8–75.0)	39.6	39.7 (38.5–40.8)	77.4	77.7 (76.7–78.7)
No	28.7	28.7 (28.6–28.8)	73.9	73.9 (73.7–74.0)	37.3	37.3 (37.2–37.4)	74.7	74.7 (74.6–74.8)

a Data were obtained from the IQVIA (formerly known as IMS Health and Quintiles) Ambulatory Electronic Medical Records (EMR) database via the IQVIA E360 Software-as-a-Service platform (9,10).

b Calculated as weight in kilograms divided by height in meters squared; overweight defined as BMI from 25.0 to 29.9.

c Calculated as weight in kilograms divided by height in meters squared; obesity defined as BMI ≥30.0.

d Based on a strict US Preventive Services Task Force recommendation-based definition: tests that were clearly labeled in the EMR as hemoglobin A_1c_, fasting plasma glucose, or oral glucose tolerance test. No oral glucose tolerance tests were found in the IQVIA database used for this analysis.

e Includes measurements labeled as hemoglobin A_1c_, fasting plasma glucose, random plasma glucose, and unspecified measures.

f We controlled for age and sex in the logistic regressions when we calculated the adjusted prevalence of blood glucose testing stratified by BMI categories. We also calculated the odds ratio of receiving blood glucose testing stratified by BMI categories; these data are available upon request from the corresponding author.

g Data on ethnicity (Hispanic origin) are not captured accurately in IQVIA electronic health records and thus these values have been suppressed.

We also identified the results of blood glucose tests using HbA_1c_ and fasting plasma glucose values. Among EMRs labeled as HbA_1c_ testing during our study period, 23.0% of tests indicated prediabetes and 5.5% indicated diabetes. Among EMRs labeled as fasting plasma glucose tests, 30.8% of tests indicated prediabetes and 5.4% indicated diabetes.

## Discussion

The prevalence of prediabetes and diabetes continues to grow in the US. Most people with prediabetes are unaware that they have the condition (17). Screening for prediabetes and diabetes has been recommended by expert groups to improve the timely provision of evidence-based diabetes prevention and treatment interventions. In 2008, the USPSTF recommended screening for type 2 diabetes in asymptomatic adults with sustained blood pressure (either treated or untreated) greater than 135/80 mm Hg (18). In the 2015 guidelines, the USPSTF recommended screening for abnormal blood glucose in asymptomatic adults aged 40 to 70 years who have overweight or obesity (7). In 2021, the USPSTF further lowered its recommended age to start screening for abnormal blood glucose in adults with overweight or obesity from 40 to 35 years (19).

Our study was based on the 2015 USPSTF recommendation to allow for the evaluation of a pre–COVID-19 pandemic period and the collection of 3 years of follow-up data to determine screening status. Our findings suggest that opportunities may exist for improvement in clinical testing practices. Among people who met USPSTF screening criteria, the prevalence of receiving a test for abnormal blood glucose within the 3-year study period (2016–2019) was 33.4% when we used the strict definition of a blood glucose test. The testing rate increased to 74.3% when we expanded the definition of blood glucose testing to include random plasma glucose and unspecified blood glucose measures (ie, the broad definition). Although the 2 definitions resulted in appreciable differences, even when using the broad definition, we found that at least 1 in 4 adults with overweight or obesity did not receive glucose testing during the 3-year period.

The USPSTF recommends that screening tests for prediabetes include the measurement of fasting plasma glucose, HbA_1c_, or an oral glucose tolerance test. We used 2 definitions of receiving a blood glucose test in our analysis to account for the fact that the labeling of laboratory tests in EMRs sometimes lacks the details needed to definitively determine whether a test was performed in a fasting state. However, when ordering metabolic tests, health care providers are often required to state whether the patient had fasted. In the future, the extraction of this information by data vendors would be helpful in conjunction with the laboratory test results to determine the fasting status of patients. In addition, blood glucose tests are included in metabolic panels, which may or may not be ordered to screen for diabetes. EMRs, including laboratory records, are not collected for research purposes, and the health care provider’s intent when ordering a laboratory test is often unknown. The ICD code that a health care provider associates with a test when ordering could provide some idea of the purpose of the test. Extracting this code with the laboratory order could provide additional information on intent. Also, processing free-text notes, such as the health care provider’s treatment plan, using natural language process or other techniques, could provide valuable insight into the provider’s intent. Currently, these data are not readily available from many data vendors. Our study also emphasizes the importance of conducting the USPSTF-recommended glucose tests to identify prediabetes and diabetes. Increased use of HbA_1c_ for glucose testing could be beneficial because HbA_1c_ is a measure of long-term blood glucose concentration and is not affected by acute changes in glucose levels caused by stress or illness. Also, HbA_1c_ measurements do not require fasting and are more convenient than a fasting plasma glucose or oral glucose tolerance test (19).

Our estimated glucose testing rate (using the strict definition) is similar to the rate found in another study (20); in that study, which used data captured in EMRs from September 2016 to August 2019, 31.3% of eligible persons received blood glucose testing. Another study (21) analyzed data from the 2013–2018 NHANES and found that among people who were eligible for glucose testing (according to the 2015 USPSTF guidelines), 65.1% reported receiving a glucose test in the previous 3 years. However, data on receipt of glucose testing in NHANES are self-reported and could be subject to recall bias.

Overweight and obesity are strong risk factors for developing prediabetes and type 2 diabetes in adults. Nationally representative US estimates for overweight and obesity prevalence are 31.1% and 42.5%, respectively, based on 2017–2018 NHANES (22). Our study found a higher rate of blood glucose testing among people with obesity than among people with overweight. One possible explanation is that primary care providers could be more aware of the need to test blood glucose among people with obesity because of the well-known association between obesity and type 2 diabetes and prediabetes. Type 2 diabetes can be prevented or delayed through lifestyle-change programs that focus on weight loss and physical activity levels (23,24). For example, the National Diabetes Prevention Program established an evidence-based lifestyle-change program to address the increasing diabetes prevalence in the US (23,24). However, as noted previously (24), the lower enrollment rates in this program among racial and ethnic minority populations present a challenge. Another study (25) highlighted the importance of increasing primary care provider awareness of, and referrals to, lifestyle-change programs. Weight-loss interventions are a core tenet of lifestyle-change programs for preventing and treating overweight, obesity, and prediabetes. In addition, nutrition therapies, weight-loss medications, and metabolic surgery can be effective for weight loss and other cardiometabolic outcomes among people with elevated blood glucose levels. These treatments may be considered according to each person’s risk (26,27).

The probability of receiving blood glucose testing was higher among men than women in our study. This result is consistent with prior research that used electronic health record data collected from November 2015 to April 2017 and found that men had 19% higher odds than women of being screened for prediabetes (28). We also found that Black patients had higher rates than White patients of receiving blood glucose testing (using the strict definition). This finding is consistent with previous research that showed higher rates of diabetes screening among Black patients (29). The American Diabetes Association (ADA) recommends diabetes screening every 3 years for all adults aged 45 years or older and for overweight adults under 45 years with an additional risk factor, such as a race or ethnicity at higher risk of diabetes (eg, African American, Asian American, Latino, Native American, Pacific Islander) (30).

When we stratified our findings by medical comorbidities, we found that the prevalence of receiving blood glucose testing was higher among adults who had hypertension than among adults with no hypertension. This finding was expected because the previous (2008) USPSTF abnormal blood glucose screening guideline (18) was based on hypertension presence (rather than overweight or obesity presence) and hypertension is identified as a risk factor for screening in the ADA guidelines (31). In addition, previous research (28) found that primary care physicians often cited hypertension as a factor they consider for diabetes screening. In our study, other risk factors, such as hyperlipidemia and depression, were also associated with receiving blood glucose testing, suggesting that screening patterns among health care providers respond to the increased risk associated with these comorbidities.

Our study adds to a growing body of research suggesting that opportunities and need exist to improve screening to detect prediabetes and diabetes. Our estimated rates of blood glucose testing determined by using the strict definition were low, and even the rates determined by using the broad definition have room for improvement. These low rates could result in missed opportunities for clinicians to offer intensive lifestyle interventions to patients with prediabetes. Adults with prediabetes who do not receive timely blood glucose testing to diagnose the condition may not have the needed information and incentive to make lifestyle changes to prevent type 2 diabetes. One recent study found that adults who were aware of having prediabetes were more than twice as likely as those who were not aware to engage in efforts to meet evidence-based lifestyle goals (4). In the absence of interventions to lower their risk of type 2 diabetes, people with prediabetes may develop diabetes sooner, have a longer exposure to dysglycemia, and develop diabetes-related complications. Improving screening rates can slow or stop the progression of prediabetes and could be considered a public health priority.

We chose to focus on the 2015 USPSTF recommendation and calculated the rate of receiving a test for abnormal blood glucose within 3 years from baseline in 2016 through 2017–2019. Had we conducted the study during 2020, our results could have been affected by changes in clinical preventive care and screening services during the COVID-19 pandemic. We plan a future study, when adequate years of follow-up data are available, to assess clinicians’ use of the newer (2021) USPSTF screening recommendations.

### Limitations

Our study was subject to several limitations. First, IQVIA data are not nationally representative, and our analysis should be replicated with population-based data sets. The actual population prevalence of glucose testing in the US could be higher or lower than our estimates because of characteristics of the EMR data set. For example, in the IQVIA data set, White adults are slightly overrepresented compared with the US population. In addition, the IQVIA data set mainly comprises larger practices and health networks, which may bias the population toward urban patients or patients with health insurance and underrepresent rural patients and patients without health insurance. Second, the IQVIA data set lacks detailed data on race and ethnicity because the information is captured in an optional, single-composite variable. Third, our study could not control for socioeconomic determinants of health, which can be associated with blood glucose testing (32,33); future studies could examine whether receiving blood glucose testing differs by socioeconomic status.

### Conclusion

In this large EMR data set we found that at least 1 in 4 adults aged 40 to 70 years with overweight or obesity did not receive glucose testing during the 3-year follow-up period. Also, we found that estimating screening rates is complicated by the lack of specificity in laboratory tests as to the intent of the health care provider and the fasting status of the patient. Extracting additional data, which may be available in the EMR, could be helpful for future surveillance purposes. Opportunities exist to improve screening among adult patients at risk for developing prediabetes and type 2 diabetes. Our findings could help inform clinical and public health efforts to improve screening for blood glucose levels among adults with overweight or obesity and strengthen efforts to prevent type 2 diabetes and diabetes-related complications.
